# Laparoscopy in Blunt Abdominal Trauma: for Whom? When?and Why?

**DOI:** 10.1007/s40719-017-0076-0

**Published:** 2017-01-28

**Authors:** Viktor Justin, Abe Fingerhut, Selman Uranues

**Affiliations:** Section for Surgical Research, Department of Surgery, Medical University of Graz, Auenbruggerplatz 29, 8036 Graz, Austria

**Keywords:** Abdominal trauma, Blunt abdominal injury, Negative laparotomy, Laparoscopy, Hollow viscus injury, Diaphragmatic injury, Mesenteric injury

## Abstract

The management of blunt abdominal trauma has evolved over time. While laparotomy is the standard of care in hemodynamically unstable patients, stable patients are usually treated by non-operative management (NOM), incorporating adjuncts such as interventional radiology. However, although NOM has shown good results in solid organ injuries, other lesions, namely those involving the hollow viscus, diaphragm, and mesentery, do not qualify for this approach and need surgical exploration. Laparoscopy can substantially reduce additional surgical aggression. It has both diagnostic and therapeutic potential and, when negative, may reduce the number of unnecessary laparotomies. Although some studies have shown promising results on the use of laparoscopy in blunt abdominal trauma, randomized controlled studies are lacking. Laparoscopy requires adequate training and experience as well as sufficient staffing and equipment.

## Introduction

The majority of fatalities worldwide in people under the age of 35 years are caused by trauma [1]. Blunt mechanisms account for 78.9 to 95.6% of injuries [2–5], with the abdomen being affected in 6.0 to 14.9% of all traumatic injuries [2, 4, 6]. Non-operative management (NOM) has been widely implemented, especially in blunt abdominal trauma. However, apart from hemodynamic instability, other specific indications call for proactive surgical diagnosis and treatment. While laparotomy has been the standard procedure for these settings, laparoscopy may be considered as an alternative. This article aims to answer the threefold question about blunt trauma laparoscopy “for whom, when, and why?” highlighting the advantages but also addressing the possible complications and pitfalls.

## Work-Up of Patients Sustaining Abdominal Trauma

Primary work-up of patients sustaining abdominal trauma relies on proper knowledge of trauma mechanism and clinical examination. The decision to operate urgently or to entertain non-operative treatment depends on the clinical presentation of the patient. Indications for urgent surgical intervention are hypotension with positive focused assessment with sonography in trauma (FAST) or diagnostic peritoneal lavage (DPL), evisceration, open pelvic fracture [7], hemodynamic instability, or diffuse peritonitis [8, 9].

**Fig. 1:**
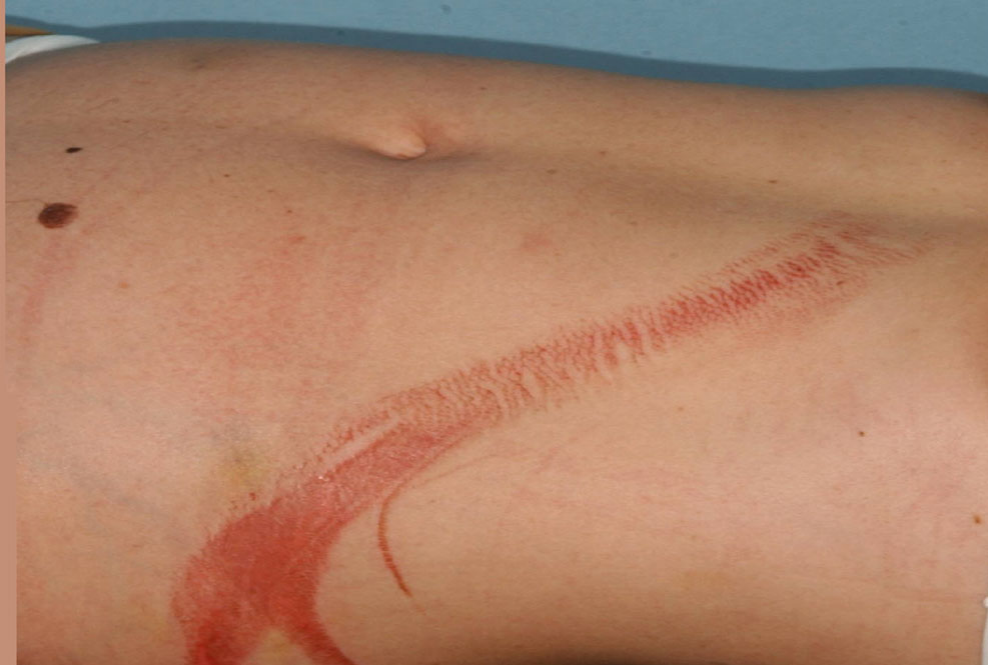
Seat belt trauma with suspected mesenteric injury on CT scan leading to laparoscopic exploration. After laparoscopic exploration confirmed the diagnosis the procedure was converted to laparotomy

The reliability of clinical examination of the abdomen can be largely compromised in case of concomitant head trauma, multiple injuries, substance abuse, and/or spinal trauma [10]. Furthermore, retroperitoneal injuries may not be associated with any relevant clinical signs. External signs such as the *seat belt sign* (Fig. 1) significantly (*p* < 0.0001) increase the likelihood of intraabdominal injuries [11, 12].

FAST has overall sensitivity and specificity rates between 43 to 86 and 96 to 99%, respectively [13–17]. Its main goal is to identify the presence of free fluid but cannot determine the source and may not detect retroperitoneal, hollow viscus, or solid organ injury without hemoperitoneum [16]. Depending on the intensity of bleeding, fluid accumulation may take some time to develop [18], making repeat examinations necessary [19]. FAST accuracy has been reported to be lower in patients with higher ISS (90.6% in ISS ≥25 vs. 97.1% in ISS <25, *p* < 0.001) [20].

DPL has a mean sensitivity of 98% (range from 90 to 100%) and a specificity of 92% (range from 73 to 100%) [21]. However, the possibility of unnecessary exploratory operations in about 15 to 20% due to the over-sensitivity and relatively low specificity of DPL has been reported [22].

Computed tomography (CT) is considered the imaging modality of choice in the hemodynamically stable and cooperative patients [23]. Holmes et al. [24] described a 0.3% missed injury rate in blunt abdominal trauma. In hollow viscus injuries (HVI), however, high rates of false-negative results (44.7 to 54.5%) have been reported by Lin et al. [25] and Bhagvan et al. [26••], independently. A large collective analysis by Fakhry et al. [27] observed normal CT scan results in 13% of patients with small bowel perforation. In blunt diaphragmatic injuries, overall sensitivity has been reported to be as low as 57% [28], with right-sided injuries being more difficult to identify [29].

In stable patients with blunt abdominal solid organ injuries, NOM is generally considered as the standard of care in the absence of indications for emergency surgery [30]. Consequent and safe implementation of NOM requires adequate staffing in terms of numbers, equipment, and skills [8]. Velmahos and colleagues [31] observed an overall NOM failure rate of 22% in blunt abdominal trauma, with marginally higher morbidity (29 vs. 45%, *p* = 0.08) as compared to successful NOM, while no difference was found in mortality rates.

Over the last decades, laparoscopy has been increasingly used as an additional tool for patients who are neither good candidates for NOM nor need an urgent laparotomy. Depending on the status of the patient and the surgical expertise of the surgeon in charge, laparoscopy offers valuable diagnostic and therapeutic possibilities.

## For Whom?

Laparoscopy should be envisioned only in patients who are hemodynamically stable and when there are no indications for trauma laparotomy. Intracranial injuries, which are associated with blunt abdominal trauma in about 46.5% [32], constitute an additional risk especially if intracranial pressure (ICP) is elevated. Indeed, abdominal insufflation and elevated intraabdominal pressure have been shown to further increase ICP, leading to potentially worsening outcome [33–35]. Other potential limitations for laparoscopy include high-grade chest trauma, preexisting intraabdominal adhesions as well as pregnancy [36].

### The Patient with Suspected Diaphragmatic Injury

**Fig. 2:**
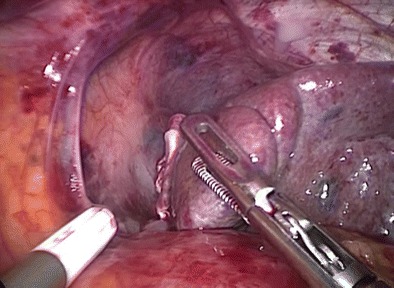
Laparoscopic exploration of the diaphragm and sealing of a superficial splenic injury

Diaphragmatic injuries (Fig. 2) mostly occur due to penetrating mechanisms and are a rare entity in blunt trauma. Two trauma database reviews from Israel [37] and the USA [38•], retrospectively analyzing more than 354,000 and 833,000 admissions, respectively, reported incidences of 0.065 to 0.148% for blunt diaphragmatic injury in all trauma patients.

Patients with blunt diaphragmatic trauma are more severely injured and have more concomitant injuries (including lesions of the thoracic aorta, lung, spleen, bladder, and pelvis) than after penetrating trauma [37, 38•]. Fair et al. [38•] described a statistically significantly higher mortality (19.8% blunt vs. 8.8% penetrating, *p* < 0.001). Whether the mortality rates were related to the diaphragmatic lesion or to concomitant injuries is not clear [39]. However, early diagnosis seems important as mortality was 25% when diagnosis was delayed compared to 3% after early diaphragm repair for penetrating injury [40].

CT scans have only limited sensitivity for diaphragmatic injury [28, 29]; thus, laparoscopy can be used to assess for and treat diaphragmatic injuries, if no other indications require laparotomy. In their 10-year experience with laparoscopy both for suspected blunt and penetrating injuries, Johnson and colleagues [41] avoided laparotomy in 89.3% applying minimally invasive diagnosis and repair.

A large analysis of the US national trauma database [42•] found laparoscopic repair of the diaphragm to be the most common therapeutic minimally invasive procedure (19.2%) in blunt and penetrating trauma. In large diaphragmatic ruptures with herniation of abdominal content into the thoracic cavity, laparoscopy alone may not be sufficient and a more complex, combined approach including double-lumen endotracheal intubation and thoracoscopy may be necessary [43–45].

### The Patient with Suspected Hollow Viscus Injury

About 0.9 to 2.5% of all trauma patients sustain HVI, most involving the small bowel [2, 5, 46]. Watts et al. [5] observed full-thickness perforations requiring urgent surgical repair in 41.5% of such patients. Morbidity was 27.6% and mortality was statistically significantly higher (19.8% with HVI vs. 12.2% without HVI, *p* < 0.001). Severe symptoms may develop after HVI, sometimes with a delay of several days after initial trauma [47]. Signs of intraperitoneal free air or free fluid on CT without detectable solid organ injury paired with signs of peritoneal irritation should prompt surgical exploration [25].

Omori et al. [48] compared 12 consecutive cases of therapeutic laparoscopy in isolated ruptured small bowel with 13 patients managed by laparotomy in a previous study. While operative time did not differ significantly (132 ± 58.7 min in laparotomy vs. 143.6 ± 27.3 min in laparoscopy, *p* = 0.296), blood loss was statistically significantly reduced (266.8 ± 277.8 mL in laparotomy vs. 57.6 ± 57.1 mL in laparoscopy, *p* < 0.05). Conversion to laparotomy was necessary in one patient, while morbidity, mortality, and duration of hospital stay were not found to be statistically significantly different.

Lin and colleagues [25] reported similar results in their case series of 135 patients, comparing two historical cohorts. Group A (62 patients, 1999–2006) was explored by laparotomy and group B (59 patients, 2007–2016) underwent exploratory laparoscopy. Conversion rate was 8.5% as opposed to a 100% laparotomy rate in the first group. While the difference in blood loss was not statistically significant, the authors observed statistically significant differences in duration of hospital stay (17.6 vs. 11.0 days, *p* < 0.001) and wound infections (16.1% [10/62] vs. 5.1% [3/59], *p* < 0.049) [25].

### The Patient with Free Fluid Without Detectable Organ Injury: Suspected Mesenteric Laceration

The hemodynamically stable patient with free fluid without signs of solid organ injury on CT [25, 49] can be managed in several ways. While in the majority of cases NOM may be sufficient, patients with suspected mesenteric or HVI (see above) must be identified.

**Fig. 3:**
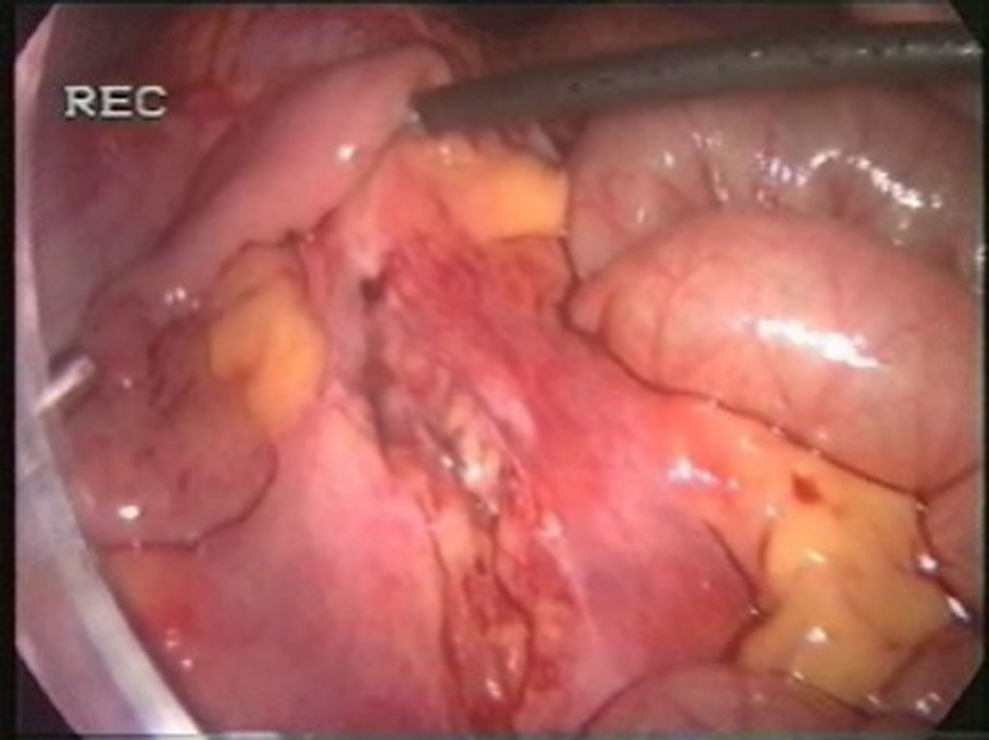
Mesenteric tear diagnosed and treated laparoscopically

**Fig. 4:**
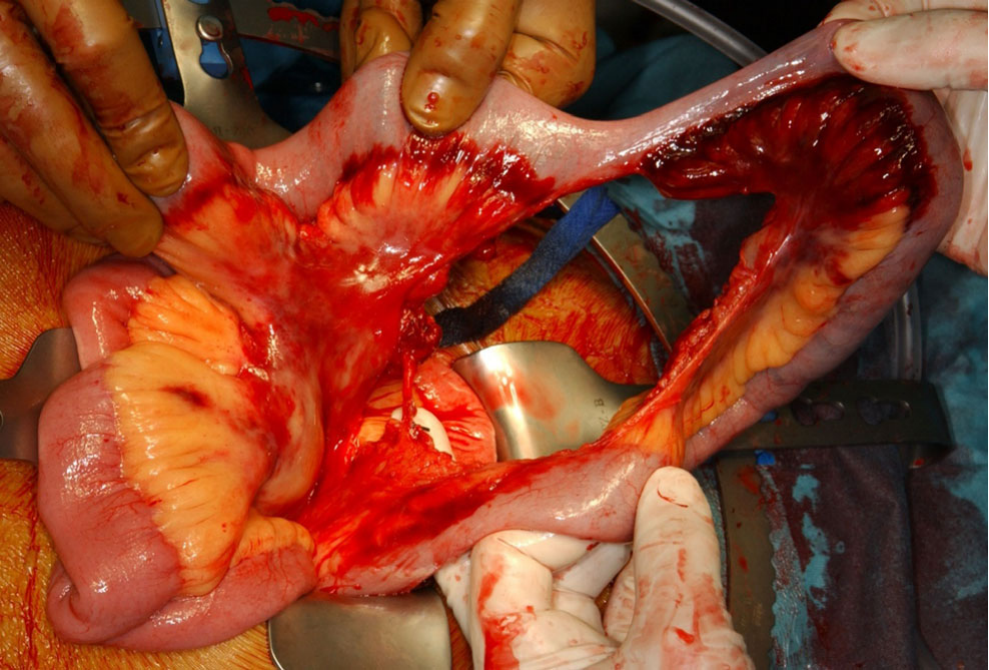
Intestinal injury first diagnosed laparoscopically

Mesenteric lacerations (Fig. 3) in blunt abdominal trauma often occur in high-speed vehicle accidents frequently due to seatbelt restraints [50•]. A retrospective report by Frick et al. [51] showed that 29.7% of mesenteric lacerations lead to bowel devascularization (Fig. 4) and consecutive complications (e.g., perforation). Thus, expectant management in these patients is risky. While CT sensitivity ranges from 75 to 99% [52–54], it may not be as accurate to determine the need for surgical intervention [52].

Laparoscopy can be used to check for bowel perfusion and resect affected bowel segments accordingly [25, 55, 56]. In cases, where sufficient blood supply is not clear, new techniques such as indocyanine green-enhanced fluorescence screening for bowel perfusion [57••] may be beneficial.

### The Unclear Abdomen

The term “unclear abdomen” includes hemodynamically stable patients with equivocal imaging studies, a substantial discrepancy between imaging studies and clinical presentation or non-specific diffuse symptoms that persist after conservative treatment [36, 58–60]. Possible causes may be preexisting pathologies (e.g., internal hernia, adhesions) unrelated to the respective trauma. In these scenarios, laparoscopy may be used to identify and treat possible preexisting conditions.

### The Patient with Complications After Initial NOM

Laparoscopy has been proven useful in the treatment of complications after NOM for severe hepatic trauma, when interventional radiology (e.g., percutaneous drainage) fails. Successful laparoscopic management of retained hemoperitoneum, infective perihepatic collections, and treatment of bile peritonitis after severe hepatic trauma initially treated by NOM have been described [61, 62] and are recommended by several guidelines [8, 9, 63]. These interventions are usually necessary 3 to 5 days post-injury [62, 64]. Since delayed operations may be considered as a failure of NOM, Letoublon et al. [64] argued that in these cases laparoscopy is “an actual part of the so-called non-operative treatment.” Similarly, the 2012 Eastern Association for the Surgery of Trauma (EAST) guidelines on NOM for blunt hepatic trauma [8] state that “Adjunctive therapies such as angiography, percutaneous drainage, endoscopy/endoscopic retrograde cholangiopancreatography and laparoscopy remain important adjuncts to nonoperative management of hepatic injuries.”

**Fig. 5:**
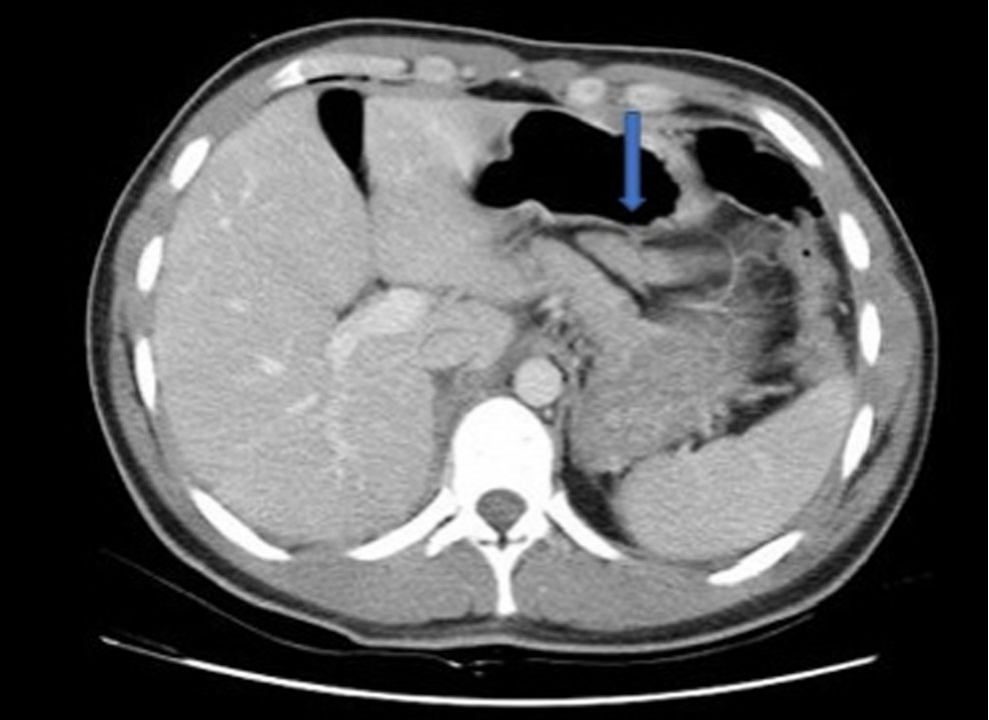
CT of a grade II pancreatic injury (blue arrow showing the injury site)

**Fig. 6:**
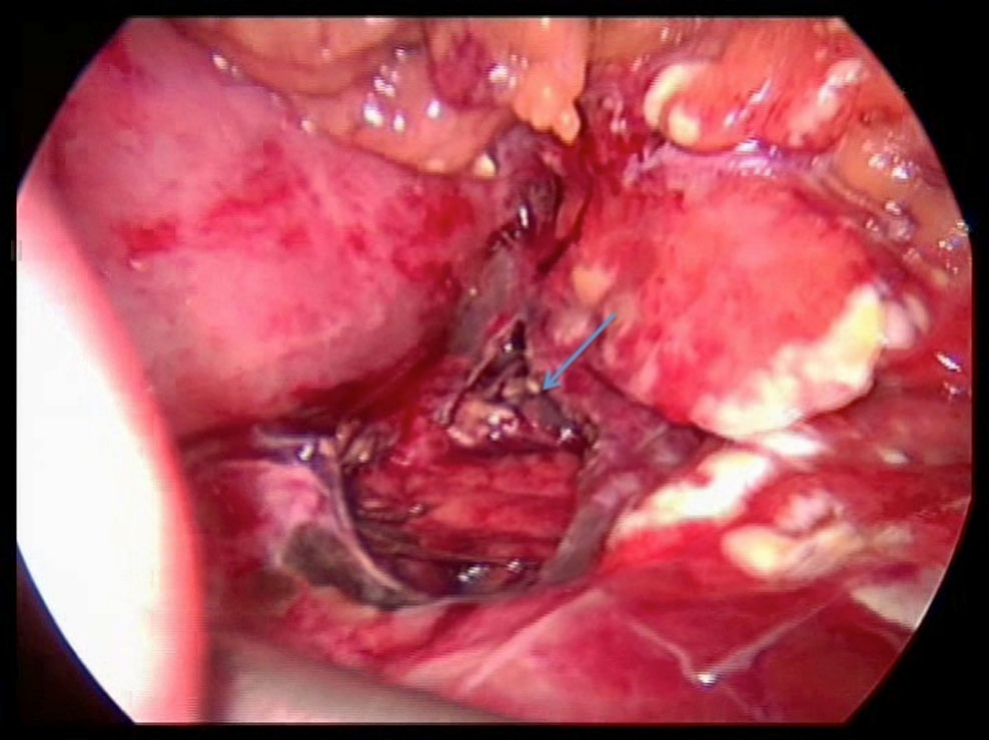
Laparoscopic debridement of grade II pancreatic injury (arrow showing the laceration)

Laparoscopy can also be of use to debride subsequent necrosis after NOM for grade I and II pancreatic injuries [65] (Figs. 5 and 6). High-grade injuries involving the pancreatic duct usually are a domain of laparotomy, although laparoscopic and even non-operative approaches have been described [66–69].

### Splenic Injuries

The treatment of splenic injuries in stable patients is non-operative, irrespective of the degree of injury [70]. In unstable patients with low-grade injuries of the spleen and if the main source of blood loss can be controlled quickly, spleen preservation can be considered [71]. Otherwise, splenectomy is the treatment of choice in hemodynamically unstable patients. Hemodynamic instability is an indication for trauma laparotomy and a contraindication for laparoscopy. Furthermore, positioning for laparoscopy in splenic surgery usually requires the patient to be in a right semilateral recumbent position, which does not allow adequate exploration of the abdomen. However, minor splenic injuries as an incidental finding during a diagnostic laparoscopy for one of the abovementioned indications can be treated laparoscopically (Fig. 2). By definition, this is an adjunct maneuver during a primarily indicated laparoscopic procedure performed for other reasons than splenic injury. Laparoscopy as a primary treatment modality in isolated splenic injuries has been reported for both splenectomy and splenic preservation with mesh splenorrhaphy in isolated cases [72–76].

### Complications of Laparoscopy in Blunt Trauma

Apart from iatrogenic access injuries, missed injuries can result in severe complications and need for delayed surgical repair. Studies from the beginning of the laparoscopic era reported missed injury rates as high as 77% [77]. More recent data, however, has revealed substantially lower missed overall injury rates ranging from 0 to 3.2% [25, 42•, 56, 78–80]. Data available specifically for blunt trauma, while scarce, have reported missed injury rates of only 0 to 0.5% [25, 56, 78, 81, 82]. Systematic approaches to abdominal exploration in minimal access surgery [83] as described previously [84] may be responsible. An open first trocar placement technique should reduce iatrogenic access injuries [85, 86].

While cases of (tension-) pneumothorax in the presence of diaphragmatic injury have been described in penetrating injuries [80, 87], to the best of our knowledge, no case reports in blunt trauma have been published. However, this complication remains possible in blunt trauma and should be entertained if patients deteriorate during laparoscopy without an explainable cause.

The possible consequences of increased ICP due to intraabdominal insufflation have been mentioned above. To the best of our knowledge, venous gas embolism [88], following laparoscopy for trauma, has not been reported so far. It has, however, been observed after ERCP for blunt hepatic trauma [89].

## When Should Laparoscopy Be Performed?

Timing of laparoscopic intervention depends on the clinical scenario. In patients presenting without indications for immediate laparotomy but need for surgical exploration (e.g., suspected mesenteric tears, HVI…) exploration should be performed after initial resuscitation is completed. Interval laparoscopy may be needed in prevention or treatment of complications after NOM.

## Why Laparoscopy?

Although NOM has reduced the rate of surgical exploration in blunt abdominal trauma with hemodynamic stability, it is still indicated in certain situations. Laparotomy as the standard approach, however, is associated with high morbidity rates up to 41.3% [90] in negative laparotomy and adds additional surgical trauma.

Several surgical societies [8, 9, 59, 63, 91–93] recommend laparoscopy for diagnosis and therapeutic intervention in selected cases as well as an approach for control of complications after hepatic NOM. However, due to the lack of randomized controlled studies and small sample sizes, these recommendations are based on low evidence levels and thus have to be interpreted accordingly [8, 59, 92, 93].

As observed by Velmahos et al. [31], more than one third of NOM failure is due to injuries, namely HVI and diaphragmatic and vascular lacerations.

Implementation of minimally invasive surgery in trauma has been reported to avoid trauma laparotomies in 7.7 to 60.7% [82, 94, 95]. Conversion rates in blunt trauma laparoscopy ranged from 8.5 to 23.8% [25, 41, 55, 82] depending on patient selection criteria. A systematic review by Zafar et al. [42•] showed an overall conversion rate of 20.2% in (blunt and penetrating) abdominal trauma.

In terms of duration of hospital stay after open vs. minimally invasive surgery, reductions have been described in laparoscopy. Comparing two groups with similar ISS after repair for blunt HVI and mesenteric injuries, Lin et al. [25] reported a mean hospital stay of 11.0 days after laparoscopy as compared to 17.6 days (*p* < 0.001) after open surgery. Similar results have been reported by Lee et al. [95] (11 vs. 21 days, *p* < 0.001) and Lim and colleagues [81] (11.5 vs. 17.6 days, *p* = 0.004).

From an economic point of view, laparoscopy might be more cost-effective than non-therapeutic laparotomy. Taner and colleagues [94] described 1.78 times higher costs in unnecessary laparotomy as compared to laparoscopy.

However, data on laparoscopy in blunt abdominal trauma are scarce and, to our knowledge, no randomized controlled trials have been published on this topic so far.

## Conclusions

In conclusion, laparoscopy in blunt abdominal trauma is safe and feasible. The prerequisites are the hemodynamic stability of the patient and surgical expertise in advanced laparoscopy. Increasing implementation of a minimally invasive approach might further reduce the gap between NOM and trauma laparotomy, thus helping to further reduce complications and longer hospital stay following unnecessary laparotomies.
